# Ingested microplastics and gastrointestinal health: a comprehensive review of their role in gut inflammation and dysbiosis

**DOI:** 10.1097/MS9.0000000000004843

**Published:** 2026-04-02

**Authors:** Abdullah Sultany, Hamid Noori, Harendra Kumar, Romaisa Kunwar, Fiza Shoaib, Saurav Kumar Mishra, Humaira Sadat Sultany, Raja Chandra Chakinala

**Affiliations:** a Department of Internal Medicine, Guthrie Robert Packer Hospital, Sayre, Pennsylvania, USA; b Nuffield Department of Clinical Neurosciences, University of Oxford, Oxford, UK; c Department of Internal Medicine, Dow University of Health Sciences, Karachi, Pakistan; d Department of Medicine and Surgery, King Edward Medical University, Lahore, Pakistan; e Department of Medicine and Surgery, Khairpur Medical College, Khairpur Mirs, Pakistan; f Department of Bioinformatics, University of North Bengal, Darjeeling, West Bengal, India; g Faculty of Medicine, Kabul University of Medical Sciences “Abu Ali Ibn Sina”, Kabul, Afghanistan; h Department of Gastroenterology, Guthrie Robert Packer Hospital, Sayre, Pennsylvania, USA

**Keywords:** gastrointestinal, gut barrier, ingested microplastics, microplastics, public health

## Abstract

Microplastics (MPs) are a new kind of pollution that may be found in food, water, and the atmosphere, raising substantial alarms about their impact on human health. This study inspects the most recent findings on the impact of MPs on gastrointestinal (GI) health, with a focus on their role in causing gut inflammation and dysbiosis. Animal studies and *in vitro* tests have indicated that MPs may disrupt the intestinal barrier, increase proinflammatory cytokines, and alter the composition of gut bacteria. These changes include a less diverse microbiota, the extinction of helpful bacteria that produce short-chain fatty acids, and the emergence of detrimental species, which cause chronic low-grade inflammation. Although there is currently limited human data, preliminary studies recommend a possible relation between MP exposure, GI problems, and inflammatory indications. The study covers the biological processes that cause these effects, how the consequences change depending on the size and composition of the particles, and the current gaps in research that may be applied to individuals. Concerning this, standardized methods to assess MP exposure in people, as well as long-term research to better understand the long-term effects of continuous MP use, are needed. Plastic pollution is worsening globally; therefore, public health must understand its impact on the gut.

## Introduction

Microplastics (MPs), defined as plastic particles smaller than 5 mm, have occurred as a significant contaminant in various ecosystems, including the food chain, over the past two decades^[^1,2^]^. These particles are produced for industrial and cosmetic uses, or are the result of the halt of larger plastic waste. MPs are easily absorbed by marine species owing to their small size, eventually entering the human gastrointestinal (GI) system via the food chain. Recent studies have revealed the presence of MPs in drinking water, seafood, vegetables, table salt, and human excrement, demonstrating that individuals are exposed to them on a global scale^[^3–5^]^. Despite considerable attention to the environmental consequences of MPs, their biological implications, particularly for human health, remain largely unknown^[^6,7^]^. The GI system is a crucial area of research, as it serves as the primary point of interaction for ingested MPs. Early findings from studies on humans and animals suggest that these particles might disrupt the immune system, alter the balance of gut bacteria, harm the intestinal barrier, and lead to inflammation in the gut^[^8,9^]^. Furthermore, MPs may affect intestinal homeostasis by transporting pathogens, heavy metals, and absorbed environmental pollutants. Understanding how MPs affect the GI tract’s function is crucial due to their significance in immune regulation, microbial symbiosis, and food absorption^[^10^]^. The implications of prolonged MP exposure may extend well beyond localized GI disease since accumulating evidence links dysbiosis and chronic gut inflammation to systemic ailments, including metabolic syndrome, autoimmune disorders, and neurodegeneration^[^11,12^].^ With an emphasis on gut inflammation and dysbiosis, this review aims to compile the most recent knowledge on MP consumption and its effects on GI health. We examine data from humans, animals, and *in vitro* trials, evaluate the underlying mechanisms, and suggest areas for further investigation. We aim to focus on future research in environmental gastroenterology and inform public health policy by highlighting the potential health consequences of MP exposure. Moreover, this study adheres to the TITAN 2025 guidelines on AI transparency^[^13^]^.

HIGHLIGHTS

- Microplastics (MPs) are raising significant concerns about their impact on human health.
- This work precisely discussed each aspect relevant to MPs for better understanding.
- Implications of prolonged MP exposure may extend well beyond localized GI disease.
- Computational approaches can be a promising lead for fundamental and translational research on the health effects of MPs.

A comprehensive literature search was conducted using PubMed, Embase, and Web of Science databases covering publications through April 2025. Search terms included: (“microplastics” OR “nanoplastics”) AND (“gastrointestinal” OR “gut” OR “intestinal”) AND (“inflammation” OR “dysbiosis” OR “microbiome” OR “barrier function” OR “health effects”), with additional searches for systemic effects (“liver” OR “kidney” OR “reproductive” OR “neurological”). Studies were included if they investigated ingested MPs and their effects on GI or systemic health, gut barrier function, inflammation, or microbiome alterations. Human studies were prioritized; animal and *in vitro* studies were included to elucidate mechanisms where human evidence was limited. Reference lists were manually screened for additional relevant studies.

### Sources and ingestion of MPs

**Figure 1.**
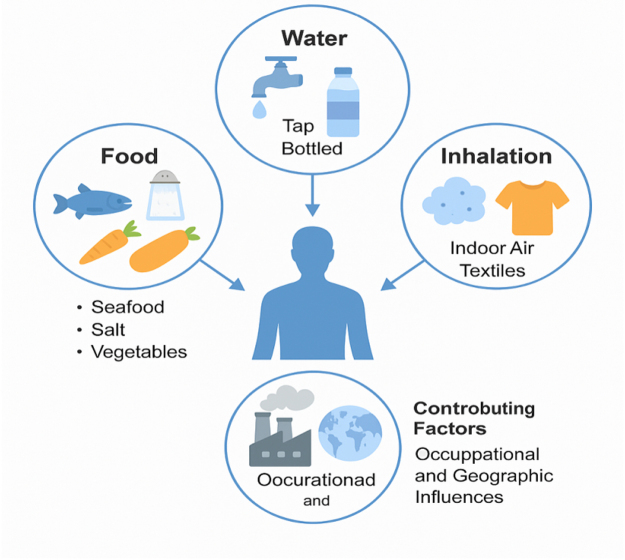
Major pathways of human MP ingestion and contributing factors.

Environmental contamination of food, water, and air enables anthropogenic particulate matter to enter the human GI tract, with ingestion of contaminated food and water constituting the principal exposure route^[^1,2^]^ as in Figure 1. MP particles have been documented across a broad spectrum of dietary items and beverages, including bottled water, shellfish, honey, fruit, vegetables, and even infant formula^[^2,3^]^. MPs accumulate in marine life, particularly filter feeders and bottom-dwelling animals, and are eventually consumed by humans. MPs may enter your body via tap or bottled water. Bottled water may contain hundreds to thousands of MP particles per liter, with many of these particles originating from packaging^[^5^]^. Food processing, storage, and packaging equipment, such as plastic containers, wraps, and utensils, may introduce MPs into food when used, heated, or stored^[^6^]^. MP exposure extends beyond dietary intake. Lifestyle habits, occupational activities, and residential environments all exert a substantial influence on an individual’s cumulative MP burden. Urban areas have higher levels of exposure due to increasing air pollution, industrial waste, and synthetic materials^[^7^]^. Individuals living in coastal or highly industrialized areas may also face increased exposure risks due to environmental pollution.

Furthermore, workers in the textile, plastic manufacturing, and recycling sectors are more likely to be exposed to MPs by ingestion and inhalation^[^8^]^. Inhalation has become a more widely accepted method of MP penetration. MPs are suspended in atmospheric dust and indoor air, particularly in confined spaces containing synthetic textiles, carpets, and furniture^[^9^]^. These particles may be inhaled and then passed by the mucociliary escalator to the oropharynx, where they are ingested and enter the digestive system^[^10^]^. This indirect absorption route demonstrates that even non-dietary exposures significantly contribute to the overall MP burden. The cumulative effects of exposure to food, water, and air pose a constant and diverse threat to human health. Although eating food is the easiest way to measure MP exposure, breathing in MPs highlights the commonality of their presence and underscores the importance of thoroughly assessing all potential ways we might be exposed to them.

### Physicochemical properties of ingested MPs

**Figure 2.**
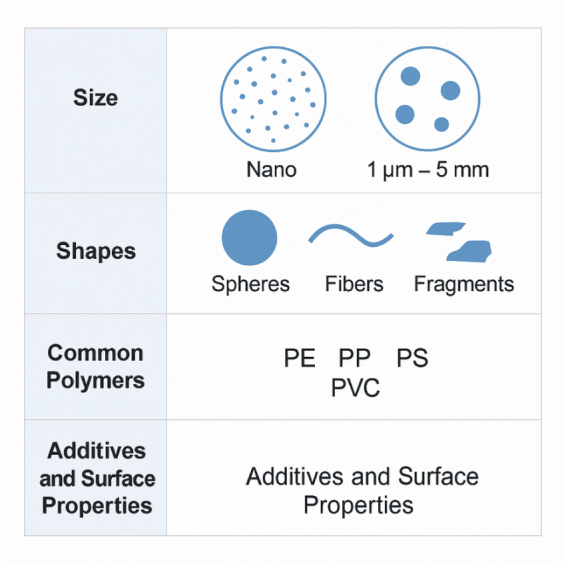
Key physicochemical features influencing MP behavior in the gut.

The detrimental health effects of MPs are heavily influenced by their diverse physicochemical properties, which determine how they interact in the GI environment (Fig. 2). Key factors include particle size, shape, polymer composition, surface properties, and the presence of additives or adsorbed toxins^[^10^]^. Size is a vital determinant of toxicity and bioavailability. MPs vary in size from a few micrometers to 5 millimeters, with smaller particles (<150 µm) more likely to pass the intestinal epithelium and reach systemic circulation via transcellular or paracellular routes^[^11^]^. Nanoplastics (<1 µm) pose greater dangers due to their potential to internalize into cells, despite limited research^[^12^]^. The shape also influences biological interactions. MPs may take several forms, including spheres, bits, threads, and films. Fibrous forms, which are often derived from synthetic fabrics, have prolonged transit times and may lodge in mucosal folds, causing chronic local discomfort^[^14,15^]^. The type of polymer influences its chemical reactivity, hydrophobicity, and degradation. Polyethylene, polypropylene, polystyrene (PS), polyethylene terephthalate, and polyvinyl chloride are among the most commonly encountered polymers in swallowed MPs. Among them, PS has been shown to induce oxidative stress and disrupt tight junctions in intestinal epithelial cells^[^16^]^. Surface properties, such as roughness, charge, and the presence of oxygen-containing functional groups, may influence how MPs interact with mucus, immune cells, and gut microorganisms^[^17^]^. Environmental aging often alters these surfaces, increasing their attraction to microbial biofilms or reactive species^[^18^]^. Many MPs also contain chemical additives, which can seep into the GI tract^[^19^]^. Furthermore, MPs may absorb external pollutants, such as persistent organic pollutants (POPs), heavy metals, and antibiotics, from their surroundings, thereby converting them into vectors for increased chemical exposure^[^20^]^. These physicochemical qualities determine how MPs interact with the gut environment, which includes bile salts, enzymes, mucus, and microbiota, possibly altering microbial populations and inducing immunological activation or dysbiosis^[^21^]^.

### MPs and gut barrier function

**Figure 3.**
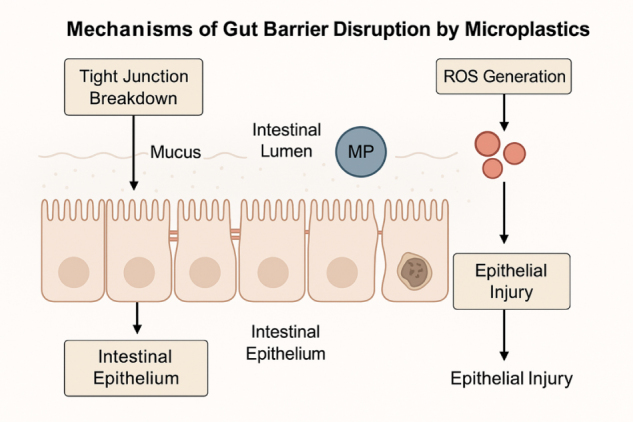
Mechanisms of gut barrier disruption by MPs. Ingested MPs disrupt intestinal homeostasis through tight junction damage, oxidative stress, and direct epithelial injury, leading to increased permeability and inflammation.

The intestinal epithelial barrier is a crucial defensive mechanism that maintains gut homeostasis and prevents hazardous substances, such as infections and toxins, from entering the bloodstream (Fig. 3). Ingested MPs have been shown to harm this barrier by mechanisms including tight junction disruption, oxidative stress, and direct epithelial injury^[^21^]^. Tight junctions are protein complexes that include claudins, occludins, and zonula occludens. They close the gap between adjacent epithelial cells and regulate paracellular permeability. Several experimental studies have shown that MPs decrease tight junction integrity. In both *in vitro* and animal investigations, PS MPs have been shown to downregulate the expression of tight junction proteins, resulting in increased intestinal permeability, also known as “leaky gut”^[^22,23^]^. This disturbance enhances the uptake of luminal antigens and poisons, potentially causing systemic inflammation^[^24^]^. Oxidative stress is another major way MPs impact the epithelial lining. When MPs, especially smaller particles with reactive surface groups, come into contact with intestinal cells, they produce an excessive amount of reactive oxygen species (ROS)^[^25^]^. This oxidative imbalance damages cellular proteins, lipids, and DNA, thereby weakening the epithelial barrier and contributing to the generation of inflammatory signals^[^26^]^. Furthermore, ROS generation activates the NF-κB pathway, leading to the release of proinflammatory cytokines such as IL-6, IL-8, and TNF-α^[^27^]^. Animal and cell-based models have provided valuable insights into understanding these consequences. In mouse studies, prolonged exposure to MPs resulted in histological signs of epithelial damage, including villus shortening, goblet cell depletion, and inflammatory cell infiltration in the lamina propria^[^28^]^. Zebrafish larvae exposed to PS MPs developed altered gut shape and apoptosis in intestinal epithelial cells^[^29^]^. *In vitro* studies using human colon epithelial cell lines (e.g., Caco-2) have yielded similar results, indicating that MPs cause suppression of tight junction proteins and barrier failure^[^30,31^]^. Overall, our results highlight the ability of MPs to alter gut barrier integrity through both physical and pharmacological mechanisms. A persistent collapse of the epithelial barrier not only predisposes individuals to local inflammation and dysbiosis but may also contribute to systemic illness through microbial translocation and prolonged immunological activation.

### MP-induced gut inflammation

**Figure 4.**
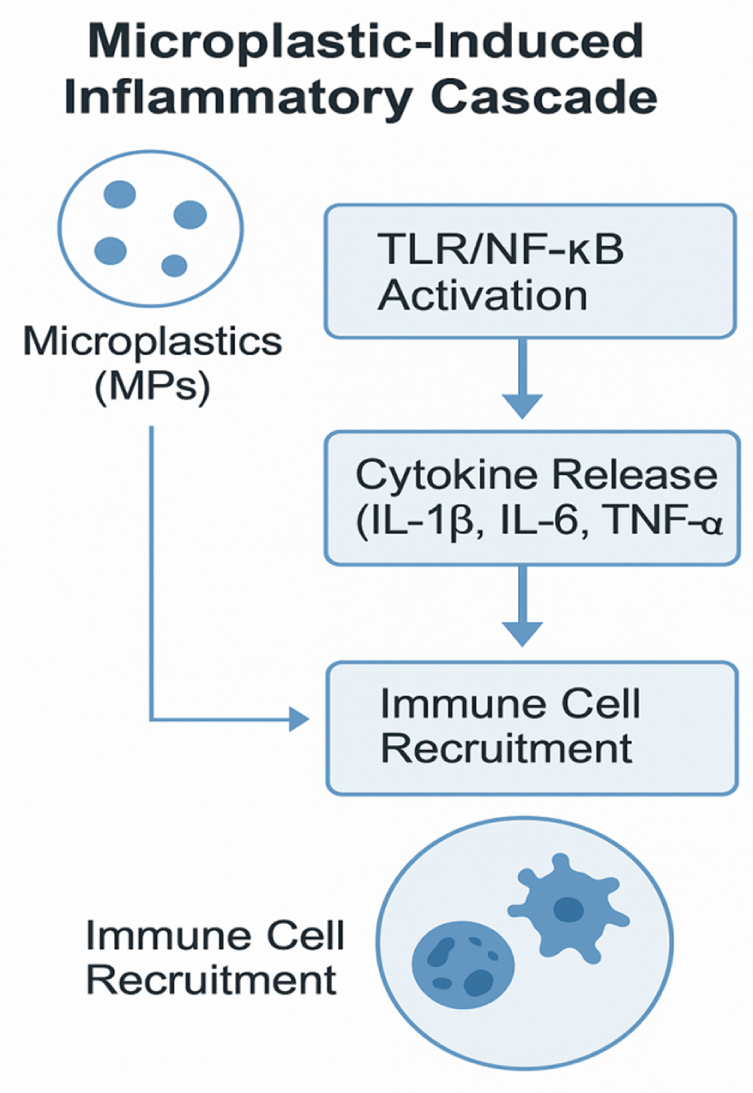
Innate immune signaling and cytokine-mediated responses induced by MPs.

Emerging research shows that MPs are key promoters of gut inflammation by altering mucosal immunity and boosting innate immune responses, as in Figure 4. These immunotoxic consequences include epithelial barrier failure, altered cytokine signaling, and a proinflammatory environment, which may contribute to the development of chronic GI disorders, such as inflammatory bowel disease (IBD)^[^31^]^. MP exposure triggers a significant innate immune response in the GI system. Pattern recognition receptors, such as Toll-like receptors on intestinal epithelial cells and resident macrophages, may detect MP-associated molecular patterns or pollutants adsorbed on their surface^[^32^]^. Upon receptor binding, subsequent signaling cascades activate transcription factors such as NF-κB and MAPK, leading to the production of inflammatory mediators^[^33^]^. Cytokine release is a defining feature of MP-induced mucosal immune response. Mice exposed to MPs exhibited elevated levels of proinflammatory cytokines, including IL-1β, IL-6, IL-8, and TNF-α, in their intestinal tissues^[^34,35^].^ These cytokines stimulate the recruitment and activation of neutrophils and macrophages, producing local tissue damage and mucosal inflammation^[^36^]^. A decrease in anti-inflammatory cytokines, including IL-10 and TGF-β, has been seen, indicating a shift towards a proinflammatory state^[^37^]^. In addition to immune cell activation, mucosal immune responses encompass changes in secretory IgA production, Paneth cell activity, and the release of antimicrobial peptides. MPs have been shown in studies on zebrafish and rats to disrupt goblet cell function and diminish mucus output, weakening mucosal defenses^[^38^]^. Chronic immunological activation by MPs may potentially impair the function of regulatory T cells (Tregs), tipping the scales toward chronic inflammation^[^39^]^. The long-term consequences of MP-induced inflammation include chronic, low-grade inflammation, which may contribute to the etiology of IBD. Animal studies have shown that recurrent exposure to MPs causes colonic hyperplasia, crypt distortion, and increased intestinal permeability, all of which are often associated with IBD^[^40^]^. While direct human evidence is lacking, mechanical links are strong. Epidemiological research has begun to investigate the connections between high ambient plastic exposure and increased incidence of IBD, although causation has yet to be established^[^41^]^. These findings contribute to the growing concern that chronic MP ingestion may cause or exacerbate gut inflammation by disrupting the epithelial barrier, activating the innate immune system, and inducing sustained cytokine dysregulation, which is central to IBD and other inflammatory gut disorders.

### Dysbiosis and microbiome alterations

**Figure 5.**
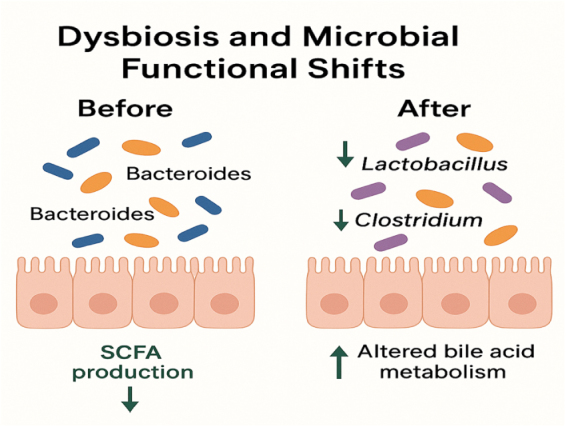
MP-associated shifts in gut microbiota and metabolic function.

The gut microbiome is important for GI and systemic health because it regulates nutrition metabolism, the immune system, and the epithelial barrier. Ingested MPs have been linked to disrupting this delicate microbial ecology, resulting in dysbiosis, a change in microbial diversity and function that may contribute to the development of illness^[^41^],^ as in Figure 5. Changes in microbiome composition are among the first and most consistent effects of MP exposure. Animal studies have revealed a decrease in microbial diversity and a shift in the relative abundance of key bacterial phyla, including a decrease in beneficial commensals, such as *Lactobacillus* and *Bifidobacterium*, and an increase in opportunistic or pathogenic taxa, such as *Escherichia* and *Clostridium* species^[^42,43^]^. In mouse models, oral treatment of PS MPs was shown to lower the Firmicutes/Bacteroidetes ratio, a well-known indicator of gut health and metabolic balance^[^44^]^. Beyond compositional modifications, functional adjustments in microbial metabolism have also been discovered. One of the most prominent anomalies is the formation of short-chain fatty acids (SCFAs). SCFAs, particularly butyrate, play a crucial role in feeding colonocytes, regulating inflammation, and maintaining barrier integrity. Studies have shown that MP-induced dysbiosis reduces SCFA levels, reducing these beneficial effects^[^45,46^]^. Furthermore, MPs affect bile acid metabolism by modifying enterohepatic circulation and microbial conversion of primary to secondary bile acids, which are known to influence lipid absorption, gut motility, and immunological signaling^[^47^]^. These differences in microbial activity and ecology are increasingly being linked to the pathogenesis of illness. MP-induced dysbiosis has been linked to higher intestinal permeability, low-grade systemic inflammation, and metabolic abnormalities in preclinical animals^[^48^]^. Alterations in microbial metabolites and immune activation may also contribute to the development of noncommunicable diseases such as obesity, type 2 diabetes, and heart disease^[^49^]^. Furthermore, decreased microbial resilience caused by chronic MP exposure may restrict the host’s ability to recover from infections or environmental shocks^[^50^]^. Preliminary human investigations indicate similar results. Fecal samples from individuals with high levels of environmental MP exposure show compositional microbial aberrations, indicating dysbiosis profiles associated with inflammatory and metabolic disorders^[^51^]^. While causation is still being investigated, the growing body of research highlights the potential of MPs to disrupt the gut microbiota in ways that go beyond localized GI symptoms and damage systemic health.

To provide a comprehensive overview of the current evidence, Table 1 summarizes the relationships between specific MP types, their associated health effects, and corresponding microbiota alterations as discussed in the preceding sections. This synthesis highlights the multifaceted impact of MP exposure on GI health and demonstrates the interconnected nature of barrier dysfunction, inflammation, and dysbiosis.

**Table 1:** Summary of MP types, associated health effects, and microbiota alterations.

MP type	GI effects	Microbiota changes
PS^[^16,22,23,44^]^	Oxidative stress induction Tight junction disruption Downregulation of tight junction proteins Increased intestinal permeability	Decreased microbial diversity Lowered Firmicutes/Bacteroidetes ratio
PE^[^29^]^	Intestinal barrier dysfunction Altered gut morphology Apoptosis in epithelial cells	Altered gut microbiota composition Affect distribution of gut microbiota
Polypropylene^[^2,5^]^	Similar effects to PE and PS Particle-dependent toxicity	Microbiota alterations Composition shifts
Polyvinyl chloride^[^4^]^	Gut barrier dysfunction Metabolism disorder	Community structure changes Microbiota dysbiosis
General MPs^[^21,24–28,30,31,34,36–38,42,43,45–47^]^	Tight junction breakdown ROS generation Epithelial injury NF-κB pathway activation Proinflammatory cytokines (IL-1β, IL-6, IL-8, TNF-α) Decreased anti-inflammatory cytokines (IL-10, TGF-β) Villus shortening Goblet cell depletion Inflammatory cell infiltration Disrupted mucus production	Decreased *Lactobacillus* and *Bifidobacterium* Increased *Escherichia* and *Clostridium* Reduced SCFA production Altered bile acid metabolism
Chronic MP exposure^[^40,48^]^	Colonic hyperplasia Crypt distortion Sustained intestinal permeability Chronic low-grade inflammation	Persistent dysbiosis Reduced microbial resilience
MPs – Systemic effects^[^52–55^]^	Hepatic inflammation Lipid metabolism disturbances Hepatic oxidative stress Renal tubular damage Altered renal biomarkers Reproductive toxicity Blood–brain barrier disruption Neuroinflammation	Gut–liver axis disruption Gut–brain axis disruption
MPs with co-contaminants^[^56^]^	Exacerbated mucosal injury Enhanced oxidative stress Increased inflammation Impaired liver function	Aggravated microbiota alterations Compounded dysbiosis

GI, gastrointestinal; IL, interleukin; MP, microplastic; NF-κB, nuclear factor kappa B; PE, polyethylene; PS, polystyrene; ROS, reactive oxygen species; SCFA, short-chain fatty acids; TGF-β, transforming growth factor-beta; TNF-α, tumor necrosis factor-alpha.

### Potential systemic effects beyond the gut

While the GI tract is the primary site of contact, ingested MPs may have systemic effects by crossing the intestinal barrier and traveling to other organs. Smaller MPs (<100 µm) and nanoplastics may reach the circulation or lymphatic system by endocytosis, leading to systemic inflammation via immunological activation^[^57^]^. Animal studies have shown that MPs are produced in the liver, leading to hepatic inflammation, disturbances in lipid metabolism, and oxidative stress^[^52^]^. MPs in the kidneys have been linked to tubular damage and altered renal biomarkers^[^53^]^. Reproductive harm has also been discovered, with studies revealing lower sperm quality, changed hormone levels, and decreased fertility in both male and female mice after chronic MP exposure^[^54^]^. Neuroinflammatory effects have been seen in animal models, suggesting that MPs may disrupt blood–brain barrier integrity and activate microglia^[^55^]^. These data indicate that MP ingestion may increase the risk of chronic illnesses such as metabolic syndrome, infertility, hepatic steatosis, and neurodegenerative disorders. While direct human data are uncommon, mechanical linkages and animal outcomes raise significant concerns and necessitate further research^[^58^]^.

### Environmental pollutants and synergistic toxicity

The ability of MPs to act as vectors for environmental contaminants raises significant concerns about their toxicity. Because of their hydrophobic surface and high surface area-to-volume ratio, MPs quickly absorb a broad range of toxicants from their surroundings, including POPs, polycyclic aromatic hydrocarbons, heavy metals, pesticides, and antibiotics^[^59^]^. Upon ingestion, these adsorbed contaminants may desorb in the GI tract, causing synergistic toxicity. Co-exposure studies have shown that MPs may increase intestinal toxin absorption, aggravate oxidative stress, and promote inflammation more than either medicine alone^[^60^]^. For example, in mouse models, MPs mixed with cadmium or bisphenol A exacerbated mucosal injury, altered the microbiota, and affected liver function^[^56^]^. This combined exposure scenario is significant in real-world circumstances, as people are seldom exposed to MPs in isolation. The synergistic toxicity of MPs and environmental pollutants may reduce bioavailability, extend retention, and disrupt detoxification processes, increasing health issues. Emerging research is increasingly focusing on describing these complex co-exposure dynamics using *in vitro* gut models, high-throughput toxicogenomic, and advanced imaging techniques. Understanding how MPs interact with other contaminants at the molecular and cellular levels is critical for developing appropriate risk assessments and regulatory policies^[^61^]^.

### Role of Artificial Intelligence and machine learning in MP research

**Figure 6.**
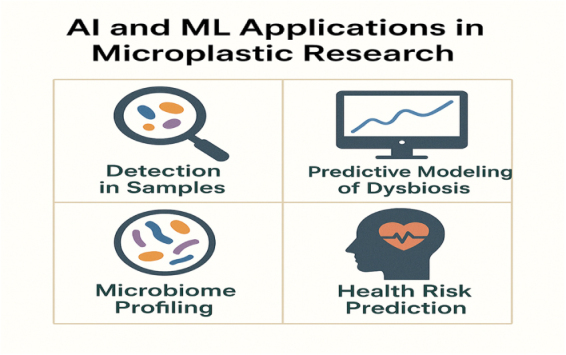
Applications of AI in MP exposure detection and health risk prediction.

The introduction of artificial intelligence (AI) and machine learning (ML) has expanded the scope of MP studies, improving detection, prediction, and understanding of their health impacts. One significant application is the detection of MPs in environmental and biological materials (Fig. 6). AI-powered image recognition systems and deep learning models have been developed to automate the categorization and quantification of MPs in complex matrices, reducing the need for human microscopy while increasing accuracy and throughput^[^1,3^]^. In the context of host-microbe interactions, ML techniques are being utilized to simulate the interaction of MPs with gut microbiota, assisting in the prediction of microbial modifications in response to different physicochemical features of MPs^[^3^]^. These models combine particle size, shape, polymer type, and surface chemical data with microbial ecology data to predict potential consequences in microbial structure and function.

Furthermore, predictive analytics using supervised ML algorithms is being investigated to evaluate the risk of gut dysbiosis and chronic illness based on exposure profiles. Researchers use animal and human datasets to train algorithms to detect early signs of MP-induced GI disease and stratify individual risk^[^4^]^. AI also plays a critical role in microbiome analysis. High-dimensional microbiome sequencing data can be analyzed using techniques such as random forest classifiers, support vector machines, and neural networks to distinguish between healthy and dysbiotic states. It opens up opportunities for personalized risk assessment, especially when combined with environmental exposure and genetic data^[^5^]^. To summarize, AI and ML offer revolutionary capabilities in MP research by simplifying detection, elucidating complex biological relationships, and enabling early prediction of health consequences. As data availability grows, these computational tools are expected to play an increasingly important role in both fundamental and translational research on the health effects of MPs^[^6^]^.

### Future perspectives and research gaps

Despite increasing evidence of MP harm, significant research gaps remain. Table 2 summarizes key studies demonstrating the GI effects of MPs across different models and exposure conditions.

**Table 2:** Key studies on MPs and GI health.

Authors (year)	Design	Population/model	Main findings	Reference
Chartres *et al*. 2024	Rapid systematic review	Human, animal	MPs “suspected” to adversely impact digestive (immunosuppression, inflammation), reproductive, and respiratory health; moderate evidence for colon cancer risk	^[^62^]^
Agrawal *et al*. 2024	Narrative review	Human, animal, *in vitro*	MPs linked to intestinal inflammation, microbiome perturbations, and IBD risk; highlights analytical challenges and research gaps	^[^63^]^
Rahman *et al*. 2021	Scoping review	Human, animal, *in vitro*	MPs exposure via ingestion, inhalation, and dermal contact; toxicity via oxidative stress, inflammation, metabolic, and neurotoxic effects	^[^64^]^
Bocker and Silva, 2025	Narrative review	Human, animal	MPs detected in feces, blood, colon, breastmilk, semen; associated with inflammation, oxidative stress, GI, and systemic disorders	^[^65^]^
Thin *et al*. 2025	Systematic review	Human, animal, *in vitro*	MPs induce gut dysbiosis, loss of beneficial bacteria, impaired SCFA production, immune modulation, and chronic inflammation	^[^66^]^
Zuri *et al*. 2023	Biomonitoring review	Human (91 studies)	MPs are detected in blood, urine, stool, lung, breastmilk, semen, placenta; associated with inflammation, oxidative stress, DNA damage, and cancer risk	^[^67^]^
Zhao *et al*. 2025	Narrative review	Human, animal, *in vitro*	MNPs cause oxidative stress, apoptosis, gut barrier dysfunction, dysbiosis, and may contribute to IBD, gastritis, and colorectal cancer	^[^68^]^
Wen *et al*. 2024	Narrative review	Human, animal, *in vitro*	Foodborne MNPs linked to gut barrier damage, inflammation, microbial alteration, and cytotoxicity	^[^69^]^
Huang *et al*. 2021	Narrative review	Human, animal	MPs disrupt gut barrier, reduce mucus, cause microbial disorders, and immune cell toxicity; evidence for oxidative damage and inflammation	^[^23^]^
Ding *et al*. 2023	Mechanistic review	Animal, *in vitro*	MPs/NPs induce oxidative stress, apoptosis, inflammation, dysbiosis, and metabolic disorders in the digestive system	^[^70^]^
de Las Hazas *et al*. 2024	Expert review	Human, animal, *in vitro*	MPs/NPs increase intestinal permeability, reduce microbiota diversity, induce inflammation, and may cause epigenetic changes	^[^71^]^
Tamargo *et al*. 2022	*In vitro* digestion model	Human colonic microbiota	MPs alter microbial community composition, promote biofilm formation, and undergo biotransformation during digestion	^[^72^]^
Nissen *et al*. 2024	*In vitro* colon model	Human colonic microbiota	Single exposure to PE/PS MPs causes overgrowth of opportunistic bacteria, reduction of beneficial taxa, and metabolic shifts	^[^73^]^

A major limitation is the scarcity of human clinical research, with the majority of accessible data coming from animal models or *in vitro* systems. Human exposure levels, biological responses, and long-term health impacts must be explored via well-designed epidemiological and interventional research^[^7^]^. Another issue is a lack of consistency in MP detection and categorization. Variability in particle separation procedures, size criteria, and polymer identification complicates cross-study comparisons. There is an urgent need for generally agreed-upon standards to ensure consistency and reliability in exposure assessments^[^8^]^. Translationally, preventative and therapeutic approaches are yet to be explored. It is crucial to explore methods to reduce MP exposure, including enhanced water filtration, alternative food packaging options, and stricter public health standards.

Furthermore, research into microbiota-targeted therapeutics, such as prebiotics, probiotics, or fecal microbiota transplantation, might provide prophylactic methods for MP-induced dysbiosis and inflammation^[^9^]^. Future studies should concentrate on vulnerable groups, such as children, pregnant women, and those with pre-existing GI disorders, who may be disproportionately affected. Interdisciplinary partnerships, encompassing environmental research, toxicology, microbiology, and AI, will be necessary to fully comprehend the health implications of MPs and inform regulatory action^[^10,16,21^]^.

### Clinical and public health risks

MPs are now recognized as a pervasive environmental contaminant with direct and indirect implications for GI and systemic health. Human exposure is nearly universal, occurring through ingestion of contaminated food and water, inhalation, and, to a lesser extent, dermal contact. MPs have been detected in human feces, blood, colon, placenta, breast milk, and other tissues, confirming their bioavailability and potential for bioaccumulation^[^65,67,74^]^.

### Clinical implications for the general population

- GI **inflammation and barrier dysfunction**: MPs can disrupt the intestinal epithelial barrier, induce oxidative stress, and promote chronic low-grade inflammation. These effects are mediated by direct physical injury, increased intestinal permeability, and immune activation, which may contribute to the pathogenesis of IBD, irritable bowel syndrome (IBS), and potentially colorectal cancer^[^23,62,63,68,70^]^.
- **Gut microbiome dysbiosis**: MPs alter the composition and metabolic function of the gut microbiota, reducing beneficial genera (e.g., *Bifidobacterium, Lactobacillus*) and promoting overgrowth of opportunistic pathogens. This dysbiosis impairs SCFA production, disrupts immune homeostasis, and is linked to metabolic syndrome, chronic inflammation, and increased susceptibility to GI and systemic diseases^[^66,72,73^]^.
- **Systemic effects**: MPs and their associated chemicals can translocate beyond the gut, accumulating in the liver, blood, placenta, and other organs. Systemic consequences include oxidative stress, DNA damage, endocrine disruption, and heightened risk for cardiovascular, reproductive, and respiratory diseases^[^65,67,75,76^]^.

### High-risk populations

- **Children**: Children are at heightened risk due to higher relative intake of food and water, frequent hand-to-mouth behaviors, and immature gut and immune barriers. MPs have been detected in placental tissue and breast milk, raising concerns about prenatal and early-life exposure, which may have long-term developmental and immunological consequences^[^65,67,76^]^.
- **Pregnant women**: Maternal exposure to MPs can result in placental transfer, with potential implications for fetal development, immune programming, and later-life disease risk. The detection of MPs in the placenta and breast milk underscores the need for targeted research and preventive strategies in this group^[^67,76^]^.
- **Individuals with pre-existing GI disease**: Patients with IBD, IBS, or other chronic GI disorders may have compromised gut barriers, making them more susceptible to MP-induced inflammation, dysbiosis, and disease exacerbation. MPs may also interact with existing environmental and genetic risk factors, amplifying disease severity^[^23,63,68^]^.
- **Occupationally exposed groups**: Workers in plastic manufacturing, recycling, and textile industries, as well as those living in urban, industrial, or coastal areas, face higher cumulative exposure through both ingestion and inhalation. These populations may experience greater bioaccumulation and health risks^[^64,65,74^]^.
- **Elderly and immunocompromised individuals**: Age-related decline in gut barrier function and immune surveillance may increase vulnerability to MP-induced GI and systemic effects, though direct evidence remains limited.

### Public health implications

- **Population-level exposure**: The ubiquity of MPs in food, water, and air means that exposure is nearly unavoidable, but the health burden is likely to be concentrated in vulnerable groups. Biomonitoring studies confirm that MPs are present in both exposed and non-exposed populations, but concentrations are higher in those with greater environmental or occupational contact^[^67,74,75^]^.
- **Disease burden and healthcare utilization**: Chronic GI inflammation, dysbiosis, and systemic effects attributable to MPs may increase the incidence and severity of IBD, metabolic syndrome, and other chronic diseases, leading to higher healthcare utilization and costs^[^62,63,66,68^]^.
- **Regulatory and preventive strategies**: There is an urgent need for standardized biomonitoring, exposure assessment, and regulatory thresholds to protect high-risk populations. Preventive strategies include improved water filtration, safer food packaging, public education, and stricter controls on plastic production and waste management^[^65,69,74,76^]^.
- **Research gaps**: Key uncertainties remain regarding the long-term effects of low-dose, chronic exposure, the impact of particle size and composition, and the potential for transgenerational effects. Longitudinal epidemiological studies and advanced experimental models are needed to clarify causality and inform evidence-based policy^[^63,66,68,71,76^]^.

### Translational relevance for clinical practice

- **Clinical awareness**: Gastroenterologists and primary care providers should be aware of the potential for MP exposure to exacerbate GI symptoms or chronic disease, particularly in high-risk patients. Consideration of environmental exposures may be warranted in the assessment and management of unexplained GI inflammation or dysbiosis.
- **Patient counseling**: High-risk individuals, especially pregnant women and parents of young children, should be counseled on practical steps to reduce exposure, such as minimizing use of plastic food containers, choosing filtered water, and avoiding highly processed foods when possible^[^65,69,74^]^.
- **Interdisciplinary collaboration**: Addressing the health risks of MPs requires collaboration between clinicians, public health professionals, environmental scientists, and policymakers to develop and implement effective mitigation strategies.

## Conclusion

MPs that are ingested offer a growing risk to GI and systemic health. Evidence from animal, *in vitro*, and early human studies suggests that they have the potential to disrupt intestinal barrier function, promote inflammation, and alter the gut microbiome – all of which are closely linked to the development of chronic disorders such as IBD, metabolic syndrome, and neuroinflammation. These effects are exacerbated when MPs act as vectors for ambient contaminants, leading to complex toxicological interactions within the GI environment. While AI and ML offer potential tools for advancing MP research, from detection to risk prediction, significant gaps remain in our knowledge, particularly in human populations. It is critical to address these gaps via standardized procedures, longitudinal research, and preventative interventions. The results of this study underscore the urgent need for a coordinated public health response to mitigate MP exposure, enhance regulatory oversight, and promote the use of safer materials. As plastic pollution spreads internationally, maintaining gut health must become a top priority in environmental health policy and research agendas.

## Data Availability

Not applicable.
